# Spatial inequalities and contextual correlates of methylphenidate dispensing in Jiangsu Province, China

**DOI:** 10.3389/fphar.2026.1926315

**Published:** 2026-08-26

**Authors:** Yao Sun, Miao Lu, Yong Zhang, Jin Xu, Feng Chen, Jing Hu, Lihua Yuan

**Affiliations:** 1 Department of Pharmacy, Children’s Hospital of Nanjing Medical University, Nanjing, China; 2 College of Hydraulic Science and Engineering, Yangzhou University, Yangzhou, China; 3 Department of Pharmacy, The First Affiliated Hospital of Nanjing Medical University, Nanjing, China

**Keywords:** contextual correlates, Getis–Ord Gi*, Jiangsu Province, LASSO, methylphenidate, SHAP, spatial inequalities

## Abstract

**Introduction:**

Understanding geographic inequalities in psychotropic medication dispensing is important for improving health equity and informing place‐based pharmaceutical management. This study characterized the spatiotemporal distribution of methylphenidate hydrochloride extended‐release tablet dispensing in Jiangsu Province, China, from 2019 to 2023 and examined its socioeconomic and education‐related correlates.

**Methods:**

Annual county‐level dispensed tablet volumes were analyzed using Getis–Ord Gi* hot‐spot analysis, standard deviational ellipses, and weighted‐centroid analysis. Prefecture‐level contextual correlates were examined using annual Lasso models interpreted with SHAP.

**Results:**

The proportion of county‐level units with zero recorded dispensing decreased from approximately 52%–36%, while the proportion with annual volumes exceeding 20,000 tablets increased from 7% to 20%. Southern Jiangsu consistently exhibited higher dispensing volumes than Northern Jiangsu, with Central Jiangsu generally occupying an intermediate position. Getis–Ord Gi* identified localized high‐value concentrations mainly in Southern and parts of Central Jiangsu. The standard deviational ellipse area increased by 23.1%, while the weighted centroid remained in south‐central Jiangsu. Within the annual Lasso models, predictive importance was allocated mainly to economic‐demographic indicators in 2019–2020 and more strongly to education‐related indicators in 2021–2023.

**Discussion:**

The findings support geographically differentiated monitoring of medication access, dispensing patterns, and prescribing quality.

## 1 Introduction

Attention-deficit/hyperactivity disorder (ADHD) is a common neurodevelopmental disorder that typically emerges during childhood and may persist into adolescence and adulthood. Methylphenidate is one of the principal pharmacological treatments for core ADHD symptoms, while extended-release formulations provide sustained therapeutic coverage and may improve adherence by reducing the frequency of daily administration (Faraone et al., 2015; Cortese et al., 2017; Wigal et al., 2014; Lee and Kim, 2025). As ADHD recognition and stimulant treatment have expanded, geographic inequalities in diagnosis, specialist access, prescribing capacity, medication availability, and continuity of care have become increasingly important concerns in pharmacoepidemiology and public health (Danielson et al., 2023).

International evidence demonstrates substantial heterogeneity in ADHD medication use across countries, age groups, and healthcare systems. A multinational study covering 64 countries and regions reported pronounced differences in ADHD medication consumption even after standardization to child and adolescent populations, suggesting that demographic structure alone cannot explain the observed variation (Chan et al., 2023). Broader international evidence has similarly documented marked cross-national differences in psychotropic medicine consumption (Brauer et al., 2021). Population-based studies from North America and Europe have identified considerable temporal and regional variation in stimulant prescribing and utilization (Raman et al., 2018; Vaddadi et al., 2021). In the United States, stimulant prescription fills have changed substantially across age groups and over time, while pronounced regional differences have been observed in the distribution of methylphenidate, amphetamine, and lisdexamfetamine (Danielson et al., 2023). German evidence has also shown substantial temporal variation in ADHD medication prescribing within a single national healthcare system (Grimmsmann and Himmel, 2021).

Studies from Asian settings further demonstrate that ADHD medication use is influenced by healthcare organization, available formulations, specialist services, and national prescribing practices. Claims-based research in South Korea documented changes in ADHD medication prescribing among children and adolescents, whereas Japanese studies reported temporal shifts in medication selection and prescribing patterns (Song and Shin, 2016; Yoshida et al., 2020). Regional variation has also been observed in psychotropic medication prescribing in Japan, highlighting the potential influence of local healthcare resources and service organization (Okui and Nakashima, 2023). Evidence from England further suggests that ADHD medication prescribing may vary according to area-level socioeconomic conditions (Khan and Hasan, 2025). Collectively, these findings indicate that ADHD medication dispensing reflects not only underlying clinical need but also differences in diagnostic practices, service accessibility, prescribing systems, treatment-seeking behavior, and local socioeconomic environments.

Previous pharmacoepidemiological studies have mainly relied on prescription databases, insurance claims, and population surveys to describe overall utilization levels, temporal trends, and age- and sex-specific prescribing patterns (Guevara et al., 2002; Raman et al., 2018). More recent research has extended this literature to prescription stimulant misuse, stimulant use disorder, and medication exposure in specific populations, including adults, secondary-school students, and the perinatal population (McCabe et al., 2023; Han et al., 2025; Nitschke et al., 2025). Spatially oriented studies have also examined geographic variation in prescription drug use and its associations with demographic, socioeconomic, and healthcare-related characteristics (Wangia and Shireman, 2013; Dijkstra et al., 2013; Vaddadi et al., 2021). Nevertheless, most existing studies have examined ADHD medications as a broad therapeutic category or have focused on national and regional averages. Fine-scale evidence concerning the spatial distribution and contextual correlates of extended-release methylphenidate as a specific formulation remains limited.

The Chinese context differs from many settings represented in the international literature because child and adolescent mental health services, specialist prescribing capacity, educational resources, and pharmaceutical supply remain unevenly distributed and are often concentrated in economically developed cities and higher-level hospitals. Although national evidence has documented increasing psychotropic medication utilization in China, geographically detailed evidence on extended-release methylphenidate dispensing remains limited (Zhang et al., 2022). A within-province analysis can reduce variation arising from national regulatory and reimbursement systems while revealing subprovincial inequalities that may be obscured by broader regional averages. Jiangsu Province is particularly suitable for such analysis because, despite its relatively strong healthcare and educational capacity, it exhibits substantial internal disparities in economic development, educational resources, and healthcare allocation (Li et al., 2020; Zhou et al., 2017; Jiang et al., 2025). Southern Jiangsu is more urbanized and has more concentrated healthcare and educational resources, whereas parts of Northern Jiangsu have more limited service capacity. These regional differences may be associated with specialist availability, prescribing capacity, medication supply, healthcare access, and school-based recognition and referral of attention and behavioral difficulties. Previous studies have also reported socioeconomic inequalities in ADHD symptoms, diagnosis, and medication use and have discussed the roles of academic pressure and parental involvement (Owens, 2021; Pearce et al., 2024).

Spatial analytical methods are increasingly used in public health and medication-use research to characterize geographic inequalities that cannot be adequately summarized by conventional descriptive statistics (Wangia and Shireman, 2013; Manda et al., 2020). Global spatial autocorrelation statistics can assess whether medication volumes exhibit an overall clustered or dispersed distribution, whereas local statistics such as Getis–Ord Gi* can identify localized concentrations of high and low values. Directional-distribution and weighted-centroid analyses can further characterize changes in spatial extent, orientation, and geographic center over time. The combined use of these methods provides a more comprehensive assessment of regional dispensing patterns than mapping alone.

Advances in machine learning and explainable artificial intelligence have also created new opportunities for examining complex predictive structures in health-related data (Atias et al., 2025; Hu et al., 2024; Yang et al., 2026). Machine-learning methods can accommodate complex relationships and correlated predictor structures, although their value depends on sample size, model specification, and appropriate validation (Breiman, 2001; Chen and Guestrin, 2016; Madakkatel et al., 2021). Regularized regression, particularly Lasso, can constrain model complexity and generate parsimonious models in small ecological datasets containing correlated contextual indicators. Random Forest, Gradient Boosting, and eXtreme Gradient Boosting provide alternative model specifications for evaluating predictive robustness. SHAP (SHapley Additive exPlanations) methods can then summarize how fitted models allocate predictive contributions across variables (Lundberg and Lee, 2017). However, feature attribution may be sensitive to correlations among predictors and to the selected algorithm; SHAP importance should therefore be interpreted as model-specific predictive attribution rather than as an independent or causal effect (Aas et al., 2021).

Accordingly, this study aimed to: (1) characterize temporal changes and county-level spatial patterns in the dispensed volume of methylphenidate hydrochloride extended-release tablets in Jiangsu Province from 2019 to 2023; (2) evaluate global spatial autocorrelation, localized high- and low-value concentrations, directional dispersion, and weighted-centroid migration; and (3) examine prefecture-level socioeconomic and education-related correlates using an interpretable and comparatively validated machine-learning framework. By integrating fine-scale spatial analysis with contextual predictive modeling, this study provides subprovincial evidence on geographic inequalities in a specific ADHD medication formulation and complements previous international research conducted primarily at national or broader regional scales.

## 2 Methods and materials

### 2.1 Hot spot analysis based on Getis-Ord Gi*

To identify statistically significant spatial clustering of methylphenidate use, we applied Getis-Ord Gi* hot spot analysis to annual county-level medication consumption. This method evaluates whether high or low values cluster spatially by comparing the local sum of neighboring observations around a focal spatial unit with the corresponding global expectation. The Gi* statistic is expressed as:

$$G_{i}^{*}=\frac{\sum_{j}w_{i,j},x_{j}\;‐\bar{X}\;\sum_{j}w_{i,j}\;}{S\sqrt{\frac{n\sum_{j}w_{i,j}^{2}‐{\left(\sum_{j}w_{i,j}\right)}^{2}}{n‐1}}}$$ (1)

where *x*_*j*_ denotes the observed medication consumption in region *j*, *w*_*ij*_ denotes the spatial weight between regions *i* and *j*, $\bar{x}$ is the global mean, *S* is the standard deviation, and n is the total number of spatial units. A significantly positive Gi* value indicates a hot spot, whereas a significantly negative value indicates a cold spot.

In this study, hot-spot analysis was implemented using the built-in Hot Spot Analysis (Getis–Ord Gi*) tool in ArcGIS. Annual spatial-unit-level methylphenidate dispensing data from 2019 to 2023 were used to identify statistically significant clusters of high and low values. A first-order Queen-contiguity spatial weights matrix was adopted for the primary analysis, and the resulting z-scores and p-values were used to classify hot spots and cold spots at the 90%, 95%, and 99% confidence levels. To assess the sensitivity of cluster identification to the spatial-weight specification, the analysis was repeated using Rook-contiguity and six-nearest-neighbor weights. Stability was evaluated using exact classification agreement and the Jaccard similarity coefficient for the identified hot-spot sets. This analysis provided a descriptive spatial basis for interpreting regional heterogeneity in methylphenidate dispensing.

### 2.2 Standard deviational ellipse and centroid migration analysis

To characterize the directional distribution and spatial dispersion of methylphenidate use, we employed the standard deviational ellipse (SDE) method. Unlike local clustering analysis, which focuses on the presence of hot and cold spots, SDE summarizes the overall spatial structure of a distribution by quantifying its weighted center, principal orientation, and dispersion. In this study, county-level centroids were used as spatial locations, and county-level medication consumption was used as the corresponding weight.

The weighted mean center of the distribution was calculated as:

$$\bar{x}=\frac{\sum_{i=1}^{n}{x_{i}w}_{i}}{\sum_{i=1}^{n}w_{i}}$$ (2)

$$\bar{y}=\frac{\sum_{i=1}^{n}{y_{i}w}_{i}}{\sum_{i=1}^{n}w_{i}}$$ (3)

where (*x*_*i*_, *y*_*i*_)denotes the coordinates of county-level analytical unit *i*, and *w*_*i*_ denotes the medication consumption assigned to county-level analytical unit *i*.

Based on the weighted mean center, the standard deviational ellipse was used to estimate the major and minor axes, ellipse area, and rotation angle of the spatial distribution. The major axis reflects the dominant direction of spatial expansion, whereas the minor axis reflects the dispersion perpendicular to that direction. Ellipse area was used as a summary measure of the overall spatial extent of methylphenidate use.

To capture temporal change, annual ellipses for 2019–2023 were compared to evaluate whether the spatial distribution of medication use expanded, contracted, or shifted directionally over time. In addition, the annual weighted mean centers were connected sequentially to depict centroid migration, thereby allowing us to assess the temporal stability or directional movement of the weighted spatial center during the study period.

### 2.3 Lasso-SHAP analysis

To examine the factors associated with regional variation in methylphenidate use, this study adopted an interpretable analytical framework combining Lasso-based feature selection and SHAP interpretation. The analysis was conducted separately for each year from 2019 to 2023, allowing comparisons of annual changes in the relative importance of socioeconomic and educational predictors.

#### 2.3.1 Variable standardization and model construction

Prior to model fitting, all variables were standardized. Lasso regression was then used to reduce redundancy among candidate predictors and identify the most informative features. The objective function of Lasso is defined as:

$$\min_{\beta_{0}\beta }\left\{\frac{1}{n}\sum_{i=1}^{n}{\left(y_{i}‐x_{ij}\beta \right)}^{2}+\alpha \sum_{j=1}^{p}\left|\beta_{j}\right|\right\}$$ (4)

where *n* is the number of samples, *p* is the number of predictors, *βj* is the regression coefficient for predictor j, and *α* is the L1 regularization parameter.

Annual predictive analyses were conducted separately for the 13 prefecture-level cities using five predictors: total number of schools, total enrollment, total school graduates, gross domestic product (GDP), and urban population. Predictive performance was evaluated using nested cross-validation. In the outer Leave-One-Out cross-validation (LOOCV), each city was held out once for testing. Within each outer training set, predictors were standardized using training-set statistics, and the Lasso regularization parameter α was selected by inner five-fold cross-validation from 50 logarithmically spaced values ranging from 10^−4^ to 10. The resulting model was then used to predict the held-out city. Performance metrics were calculated from the pooled out-of-sample predictions.

#### 2.3.2 Model evaluation

Model performance was evaluated using LOOCV. The predictive accuracy of the annual Lasso models was assessed using root mean square error (RMSE), mean absolute error (MAE), and the coefficient of determination *R* ^*2*^. These metrics were used to compare model performance across years. Because the models were fitted separately for each year, the resulting performance statistics reflected the annual predictive capacity of the selected socioeconomic and educational indicators for county-level methylphenidate consumption.

#### 2.3.3 SHAP interpretation and robustness assessment

To improve interpretability, SHAP was used to quantify the contribution of each predictor to model output. SHAP is based on Shapley value theory and measures the marginal contribution of a feature to the predicted outcome for each observation. The SHAP value for feature *j* is defined as:

$$\phi_{j}=\sum_{S\subseteq F\left\{j\right\}}\frac{\left|S\right|!\left(\left|F\right|‐\left|S\right|‐1\right)!}{\left|F\right|!}\left[f_{S\cup \left\{j\right\}}\left(x_{S\cup \left\{j\right\}}\right)‐f_{S}\left(x_{S}\right)\right]$$ (5)

where *F* denotes the full set of features, *S* denotes a subset of features excluding feature *j*, and *f*(·) represents the prediction function of the fitted model.

After predictive performance had been evaluated using nested LOOCV, each annual Lasso model was refitted to the complete annual dataset for *post hoc* interpretation. Predictor standardization and selection of the regularization parameter *α* were repeated using the complete dataset. SHAP values were then calculated for the refitted models, and mean absolute SHAP values were used to derive annual feature-importance rankings and normalized contribution shares for the primary predictor set. Separate exploratory Lasso models were also fitted using decomposed socioeconomic and education-related indicators. The SHAP results were used solely to interpret the fitted predictive structure rather than to estimate out-of-sample performance. Given the strong correlations among predictors, feature contributions were interpreted as model-specific allocations of predictive information rather than independent or causal effects. Comparative validation was conducted using Random Forest, Gradient Boosting, and XGBoost models fitted to the same annual outcomes and primary predictor sets. All models were evaluated using identical outer LOOCV splits, and predictive performance was compared using R^2^, RMSE, and MAE. Because the annual datasets contained only 13 prefecture-level cities, the alternative models used prespecified parsimonious settings to limit overfitting.

To assess the sampling stability of broader predictor domains, 1,000 bootstrap samples were generated separately for each year by resampling the 13 cities with replacement. Predictors were restandardized within each bootstrap sample, and Lasso models were fitted using the *α* selected from the complete annual dataset. The absolute standardized coefficients were normalized to sum to one and then aggregated into an economic-demographic group comprising GDP and urban population and an education-related group comprising total number of schools, total enrollment, and total school graduates. Bootstrap medians, percentile-based 95% intervals, and the proportion of bootstrap replications in which education-related importance exceeded economic-demographic importance were reported. These bootstrap results were treated as exploratory assessments of sampling stability rather than as evidence of independent or causal effects.

### 2.4 Study area and data

Jiangsu Province is located in the eastern coastal region of China (30°45′-35°20′N, 116°18′-121°57′E). It administers 13 prefecture-level cities and 95 county-level analytical units and had a resident population of approximately 84.56 million in 2023. As one of the most economically developed provinces in China, Jiangsu exhibits substantial intra-provincial disparities in economic development, educational resources, and healthcare provision, making it an appropriate setting for examining regional heterogeneity in medication use. The outcome variable of this study was the annual county-level consumption of methylphenidate hydrochloride extended-release tablets in Jiangsu Province from 2019 to 2023. Medication use was measured as the annual dispensed tablet volumes in each county-level analytical unit.

The medication data were obtained from the health data management platform established under the Drug and Medical Device Administration Center of the Jiangsu Provincial Health Commission. Socioeconomic and educational explanatory variables for the same period were derived from the Jiangsu Statistical Yearbook for each year. Based on the analytical framework of this study, the main candidate variables included urban population, gross domestic product (GDP), enrollment, number of graduates, and number of schools. Additional decomposed indicators were also considered in supplementary analyses, including the primary, secondary, and tertiary industry sectors, as well as school-type-specific enrollment, graduates, and number of schools. The three education-related variables were aggregated across primary, regular secondary, and senior secondary schools. They were treated as contextual indicators of school-system scale rather than direct measures of teacher quality, academic pressure, ADHD prevalence, or treatment need.

Geographic names and identifiers were standardized before the annual medication, boundary, and contextual datasets were linked. Records were checked for duplicated unit-year observations, nonnumeric entries, impossible negative values, unmatched geographic identifiers, and missing fields. No imputation was required because no missing values remained after linkage in the variables used for analysis. Zero tablet volumes were retained as valid observations. Potential extreme values were checked against the source records, and no valid observation was excluded solely because of its magnitude.

## 3 Results

### 3.1 Temporal trends, spatial patterns, and regional heterogeneity

**FIGURE 1:**
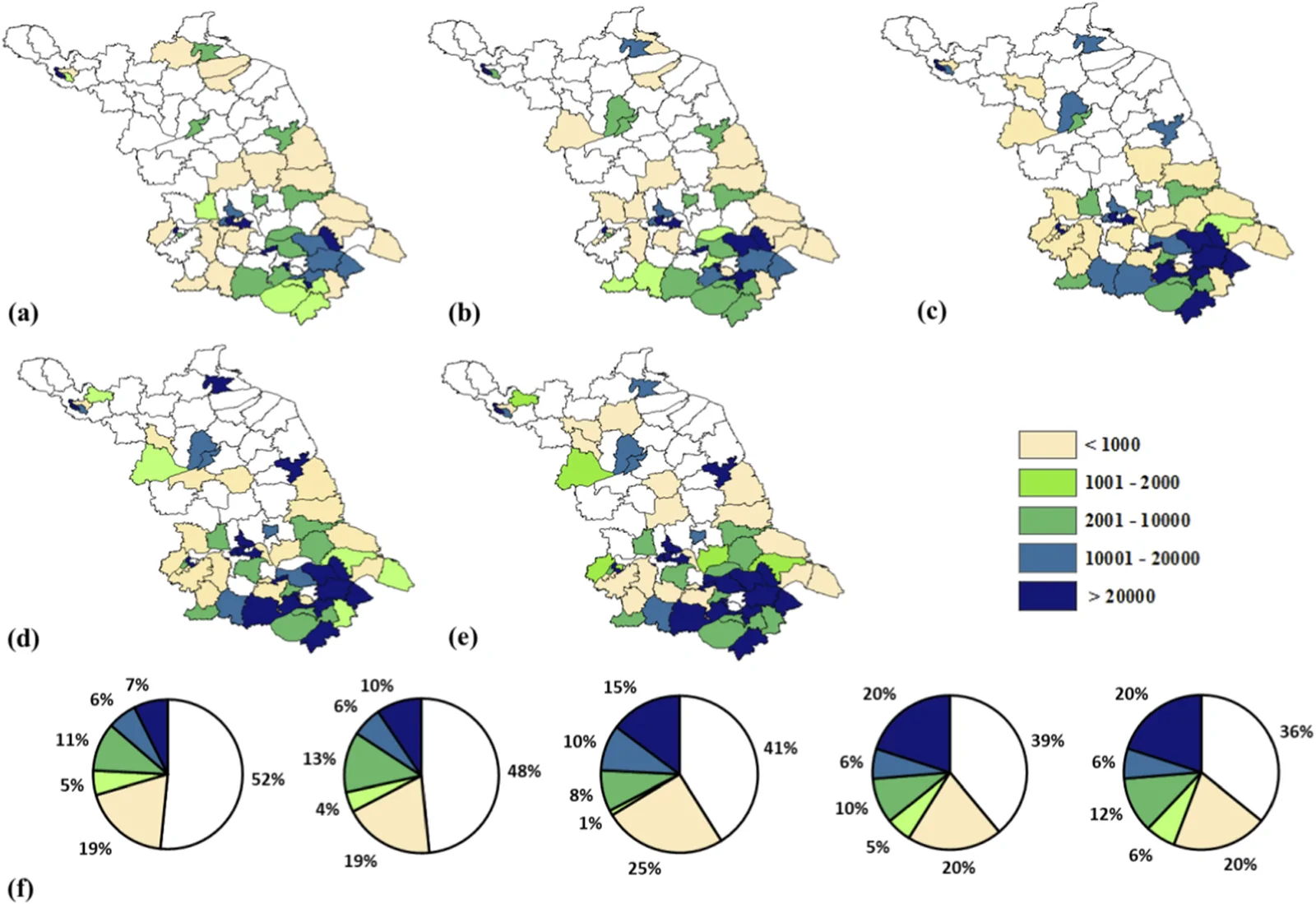
Spatial distribution and proportional composition of county-level methylphenidate dispensed tablet volume in Jiangsu Province, 2019–2023. **(a)** 2019. **(b)** 2020. **(c)** 2021. **(d)** 2022. **(e)** 2023. **(f)** Proportions of different grades (2019 2020 2021 2022 2023).

**FIGURE 2:**
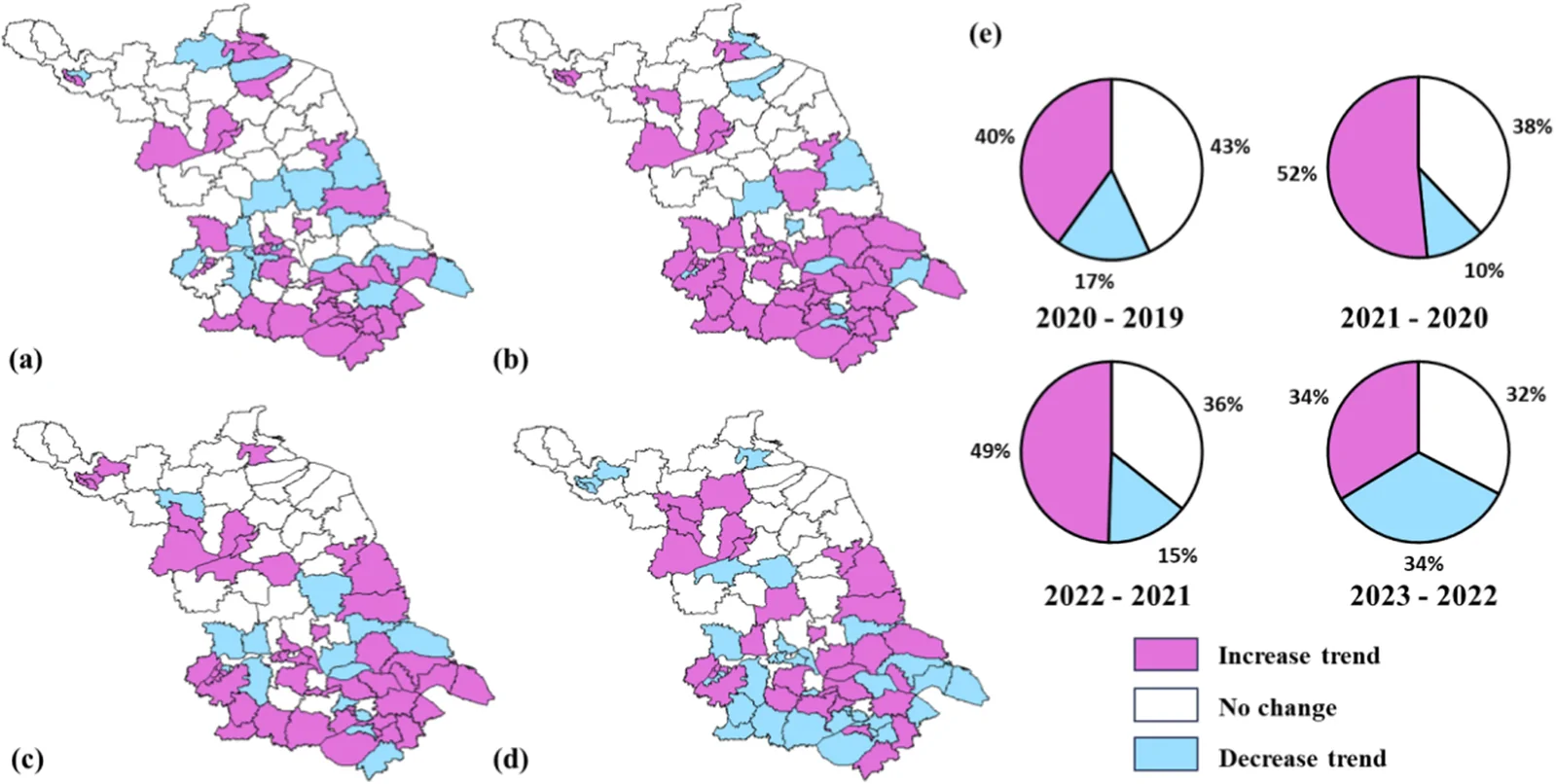
Annual changes and proportional composition of county-level methylphenidate dispensed tablet volume in Jiangsu Province, 2019–2023. **(a)** 2020-2019. **(b)** 2021-2020. **(c)** 2022-2021. **(d)** 2023 2022. **(e)** proportions of different grades.

The county-level distribution of methylphenidate hydrochloride extended-release tablet volume in Jiangsu Province from 2019 to 2023 is shown in Figure 1. Dispensed tablet volumes were generally higher in southern Jiangsu, particularly in county-level areas within Nanjing, Wuxi, and Suzhou. Over time, the geographic coverage of recorded dispensing expanded, while the number of county-level units with zero recorded volume decreased. The distribution of county-level units across dispensing-volume categories also shifted upward during the study period (Figure 1f). The proportion of units with zero recorded tablet volume decreased from approximately 52% in 2019 to 36% in 2023, whereas the proportion with annual volumes exceeding 20,000 tablets increased from 7% to 20%. These findings indicate that recorded dispensing became geographically more widespread and that a growing proportion of county-level units moved into higher-volume categories. Figure 2 presents annual changes in county-level dispensed tablet volume. Between 2019 and 2020, the proportions of units showing increases and decreases were relatively similar. Increasing volumes became more common between 2020 and 2022 and were concentrated mainly in parts of southern and central Jiangsu. More than half of the county-level units recorded an increase between 2020 and 2021. In contrast, the proportion showing decreased volume reached 34% between 2022 and 2023, indicating a partial attenuation of the preceding expansion.

Regional heterogeneity was examined by grouping county-level units into Southern, Central, and Northern Jiangsu according to their prefecture-level affiliation. The three groups contained 39, 19, and 37 analytical units, respectively. Annual differences were evaluated using Kruskal–Wallis tests, followed by pairwise Mann–Whitney U tests with Holm adjustment. Tablet-volume distributions differed significantly among Southern, Central, and Northern Jiangsu in every study year, with all Kruskal–Wallis tests yielding p < 0.005. Southern Jiangsu consistently had the highest mean and median volumes, Central Jiangsu generally occupied an intermediate position, and Northern Jiangsu had the lowest values and the largest proportion of units with zero recorded dispensing. For example, the proportion of zero-volume units in Northern Jiangsu decreased only from 70.3% in 2019 to 62.2% in 2023, compared with a decline from 38.5% to 15.4% in Southern Jiangsu. Pairwise tests confirmed that Southern and Northern Jiangsu differed significantly in every year after Holm adjustment, whereas differences involving Central Jiangsu were less consistent (Supplementary Table S1).

### 3.2 Spatial clustering, directional dispersion, and centroid migration

Before the local hot-spot analysis, Global Moran’s I was used to test whether the original methylphenidate tablet volumes exhibited province-wide spatial autocorrelation. The primary spatial-weights matrix was based on first-order Queen contiguity and was row-standardized. Statistical significance was assessed using 999 random permutations and two-sided permutation p-values. First-order Rook contiguity and six-nearest-neighbor (KNN6) weights were used as alternative specifications. Under the primary Queen-contiguity specification, Global Moran’s I did not indicate statistically significant spatial autocorrelation during any year from 2019 to 2023 (Supplementary Figure S1; Supplementary Table S2). The same conclusion was obtained under the alternative Rook and KNN6 spatial-weight specifications. Thus, the absolute tablet volumes did not exhibit a statistically significant overall clustered or dispersed pattern across the complete study area.

**FIGURE 3:**
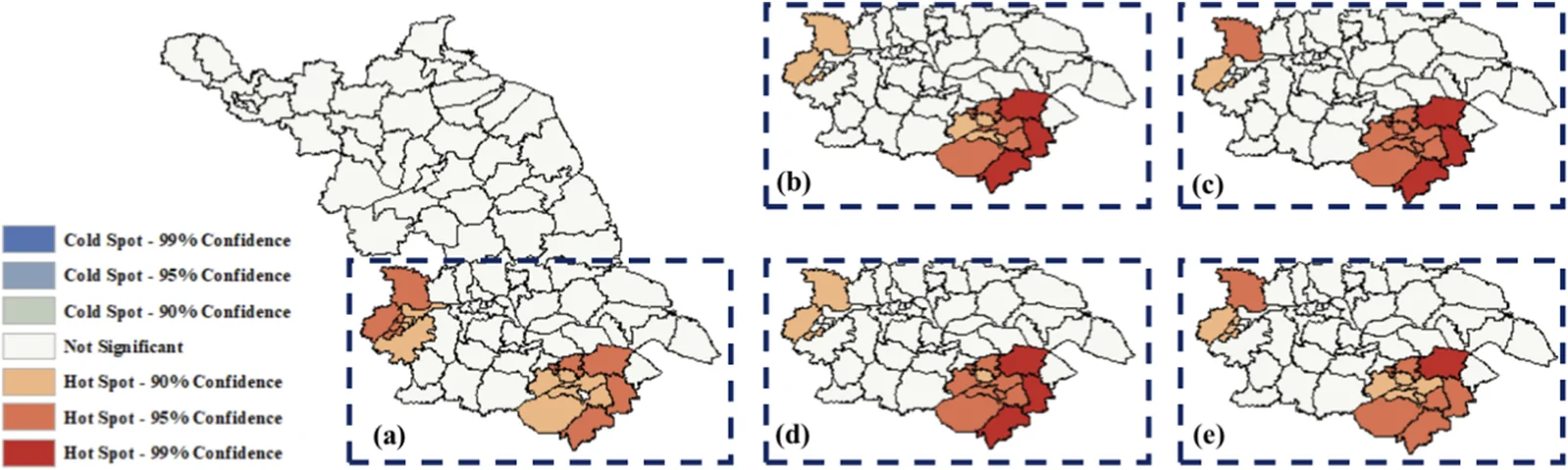
Local Getis–Ord Gi* hot- and cold-spot patterns of methylphenidate dispensed tablet volume in Jiangsu Province, 2019–2023. The analysis was based on a first-order Queen-contiguity spatial weights matrix. Positive Gi* z-scores indicate hot spots and negative z-scores indicate cold spots. The 90%, 95%, and 99% categories correspond to two-sided thresholds of ∣z∣ ≥1.645, ∣z∣ ≥1.960, and ∣z∣ ≥2.576, respectively. **(a)** 2019. **(b)** 2020. **(c)** 2021. **(d)** 2022. **(e)** 2023.

Although Global Moran’s I was nonsignificant, the Getis–Ord Gi* analysis identified localized concentrations of high tablet volume under the primary Queen-contiguity specification (Figure 3). This apparent difference is statistically plausible because Global Moran’s I summarizes average spatial dependence across the entire study area, whereas Gi* identifies localized clusters that may occur within an otherwise globally random distribution. Accordingly, the areas shown in Figure 3 were interpreted as local high-value concentrations rather than as evidence of a province-wide clustering process. Based on the Getis–Ord Gi* analysis under the primary Queen-contiguity specification, statistically significant hot spots were concentrated mainly in Southern and parts of Central Jiangsu, whereas no statistically significant cold spots were identified in any study year. Eight county-level units were classified as hot spots in 2019, nine in each year from 2020 to 2022, and eight in 2023. The confidence levels of the identified hot spots varied across years, with several county-level units reaching the 99% confidence level in 2021 and 2023. These results indicate persistent localized concentrations of high dispensed tablet volume but do not provide evidence of statistically significant low-value clusters in Northern Jiangsu.

Hot-spot stability was assessed using Rook and KNN6 weights, with consistency evaluated by exact classification agreement and Jaccard similarity of the identified hot-spot sets. Queen and Rook produced identical classifications in all years. Agreement between Queen and KNN6 ranged from 84.2% to 87.4%, whereas hot-spot Jaccard similarity ranged from 0.333 to 0.467 because KNN6 delineated broader clusters. No cold spots were identified under any of the three spatial-weight specifications (Supplementary Table S3). Overall, the locations of the main high-value areas were robust to the choice of contiguity specification, whereas cluster extent remained sensitive to the definition of spatial neighborhoods.

**FIGURE 4:**
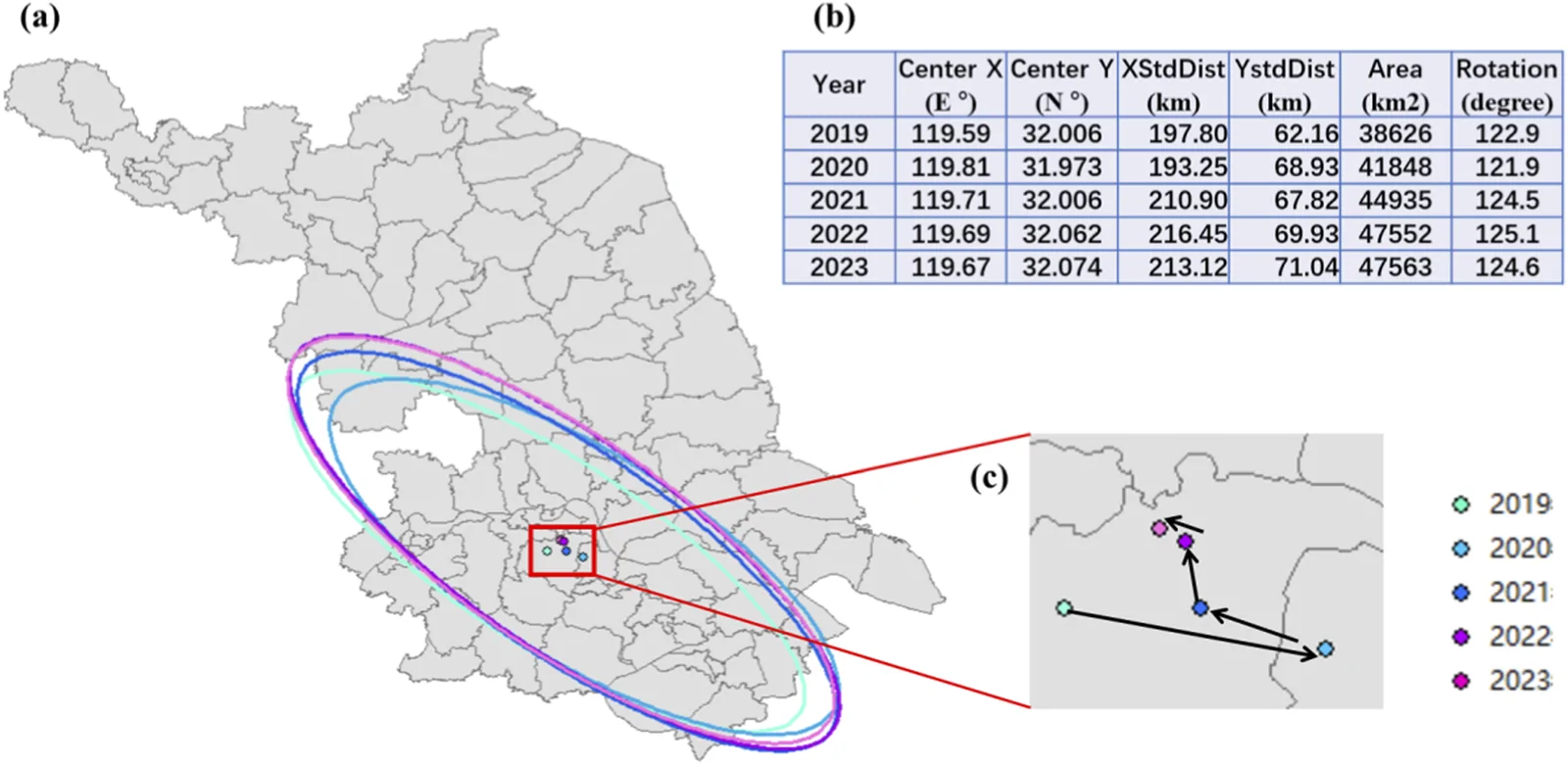
Standard deviational ellipses of methylphenidate dispensed tablet volume **(a)**, annual ellipse parameters **(b)**, and weighted-centroid migration **(c)** in Jiangsu Province, 2019–2023.

Figure 4 presents the standard deviational ellipse (SDE) of methylphenidate dispensed tablet volume in Jiangsu Province from 2019 to 2023 (Figure 4a) and the sequential migration of the tablet-volume-weighted centroid (Figure 4b). The SDE results indicate a gradual expansion in the spatial dispersion of methylphenidate use during the study period. The standard deviation along the east-west axis increased from 197.80 km in 2019 to 213.12 km in 2023, while the standard deviation along the north-south axis increased from 62.16 km to 71.04 km. Correspondingly, the ellipse area expanded from 38,626 km^2^ to 47,563 km^2^, indicating an overall enlargement of the spatial extent of medication use.

Meanwhile, the ellipse rotation angle remained relatively stable, ranging from approximately 122.9°to 124.6°, which indicates a persistent northeast-southwest orientation of the principal spatial distribution axis. The annual weighted centroid showed a modest southeastward shift between 2019 and 2021, moving from 119.59° E, 32.006° N to 119.71° E, 32.006° N, followed by a slight westward adjustment in 2022 and 2023. Overall, although methylphenidate use expanded spatially over time, the weighted spatial center remained within the south-central part of Jiangsu throughout the study period.

### 3.3 Predictive importance and comparative model performance

To examine the contextual correlates of regional variation in methylphenidate dispensing, SHAP was used to interpret annual Lasso models fitted separately for 2019–2023. Predictive importance was quantified using mean absolute SHAP values, and relative contribution rates were calculated by dividing each predictor’s mean absolute SHAP value by the summed values of all predictors within the corresponding annual model. Standardized prefecture-level values of annual dispensed tablet volume and the five primary predictors are shown in Supplementary Figure S2. Spearman and Pearson correlation analysis indicated substantial interdependence among the socioeconomic and education-related predictors (Supplementary Figure S3). Although Lasso constrained model complexity, it did not eliminate predictor dependence; therefore, the resulting importance values were interpreted as model-specific predictive attributions.

Comparative out-of-sample performance is summarized in Table 1. Based on pooled outer-LOOCV predictions, Lasso achieved the highest R^2^ and the lowest RMSE and MAE in every year. Lasso R^2^ increased from 0.716 in 2019 to 0.993 in 2023. The corresponding R^2^ ranges were 0.358–0.533 for Random Forest, 0.420–0.605 for Gradient Boosting, and 0.357–0.524 for XGBoost. These results indicate that the parsimonious regularized linear model provided the strongest predictive performance for the available annual datasets.

**TABLE 1:** Comparative LOOCV performance of Lasso and alternative machine-learning models, 2019–2023.

Year	Model	N	RMSE	MAE	LOOCV R2	Spearman observed-predicted	Median selected α
2019	Lasso (nested LOOCV)	13	0.168	0.129	0.716	0.852	0.00041
2019	Random forest	13	0.253	0.157	0.358	0.758	​
2019	Gradient boosting	13	0.241	0.153	0.420	0.709	​
2019	XGBoost	13	0.250	0.151	0.377	0.692	​
2020	Lasso (nested LOOCV)	13	0.151	0.112	0.764	0.775	0.00339
2020	Random forest	13	0.242	0.170	0.398	0.604	​
2020	Gradient boosting	13	0.230	0.167	0.455	0.720	​
2020	XGBoost	13	0.250	0.185	0.357	0.709	​
2021	Lasso (nested LOOCV)	13	0.118	0.094	0.850	0.786	0.00010
2021	Random forest	13	0.234	0.159	0.416	0.522	​
2021	Gradient boosting	13	0.229	0.163	0.441	0.593	​
2021	XGBoost	13	0.224	0.151	0.464	0.703	​
2022	Lasso (nested LOOCV)	13	0.090	0.072	0.913	0.868	0.00066
2022	Random forest	13	0.216	0.146	0.496	0.687	​
2022	Gradient boosting	13	0.202	0.144	0.561	0.621	​
2022	XGBoost	13	0.222	0.158	0.471	0.703	​
2023	Lasso (nested LOOCV)	13	0.027	0.020	0.993	0.989	0.00010
2023	Random forest	13	0.221	0.157	0.533	0.714	​
2023	Gradient boosting	13	0.203	0.149	0.605	0.692	​
2023	XGBoost	13	0.223	0.156	0.524	0.720	​

N = 13 prefecture-level cities per year. For Lasso, predictive performance was evaluated using nested cross-validation. In each outer LOOCV, iteration, one city was held out for testing, while α, the L1 regularization parameter, was selected within the remaining 12 cities using inner five-fold cross-validation over 50 logarithmically spaced values from 10−4 to 10. The reported median α is the median of the 13 values selected across the outer folds. Random Forest, Gradient Boosting, and XGBoost used prespecified conservative hyperparameters and the same outer LOOCV splits. RMSE, MAE, R2, and Spearman ρ were calculated from the 13 pooled out-of-sample predictions. RMSE and MAE are unitless because the outcome was normalized to a 0–1 scale separately within each year. R2 therefore represents outer-LOOCV performance rather than training performance. A dash indicates that α was not applicable.

**FIGURE 5:**
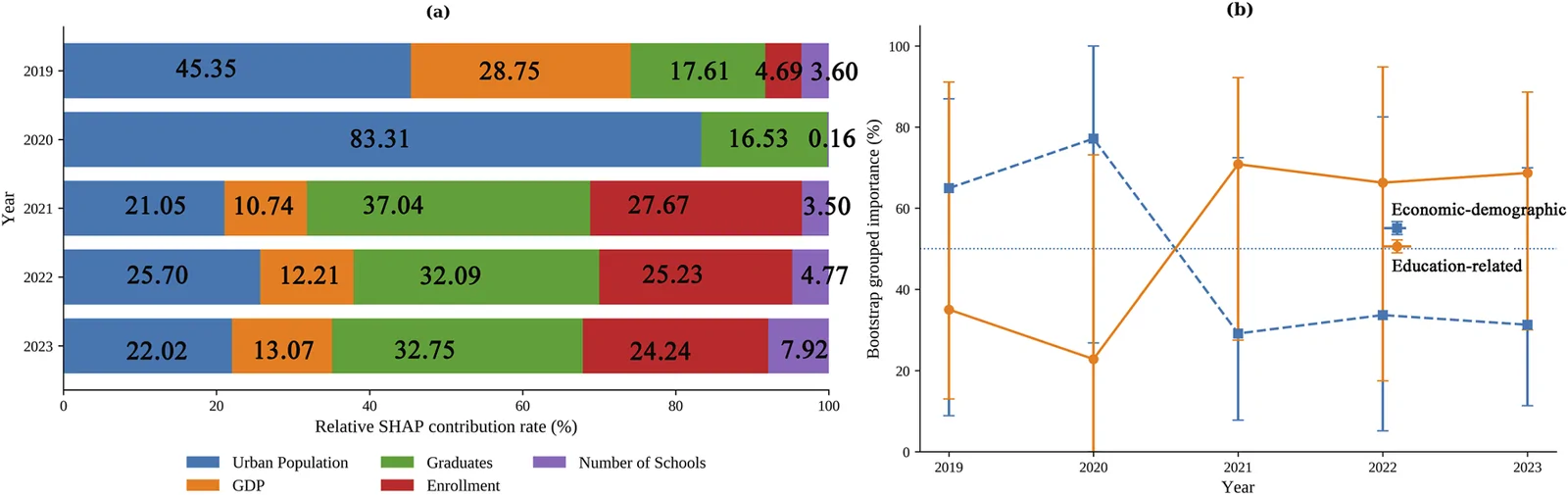
Temporal variation and stability of Lasso-based predictive attribution, 2019–2023. **(a)** Relative mean absolute SHAP contribution rates from the annual full-data Lasso models. **(b)** Bootstrap medians and percentile 95% intervals of grouped relative absolute standardized Lasso coefficients.

Within the Lasso-SHAP framework, the relative contribution rates showed temporal variation in the allocation of predictive importance (Figure 5a). In 2019, Urban Population accounted for 45.35% of total mean absolute SHAP importance and GDP accounted for 28.75%, whereas Graduates, Enrollment, and Number of Schools accounted for 17.61%, 4.69%, and 3.60%, respectively. In 2020, Urban Population accounted for 83.31% of total model-attributed importance. From 2021 onward, larger shares were allocated to education-related variables. Graduates and Enrollment accounted for 37.04% and 27.67% in 2021, 32.09% and 25.23% in 2022, and 32.75% and 24.24% in 2023, respectively. By 2023, these two variables together represented 56.99% of total Lasso-SHAP importance, compared with 35.09% for Urban Population and GDP combined.

The grouped coefficient-bootstrap analysis provided an additional assessment of the sampling stability of the two predictor domains (Figure 5b). The median economic-demographic importance share was 64.9% in 2019 and 77.2% in 2020, whereas the median education-related share was 70.9%, 66.3%, and 68.7% in 2021–2023, respectively. The bootstrap probabilities that education-related importance exceeded economic-demographic importance were 0.935, 0.814, and 0.942 in 2021–2023. However, the percentile intervals were wide, indicating substantial uncertainty associated with the small annual samples and correlated predictors.

Exploratory decomposed analyses were conducted for industrial structure and education-related indicators (Table 2). Within the industrial-structure models, the tertiary industry received the largest relative SHAP contribution in every year, ranging from 67.48% to 100.00%. Within the school-level enrollment models, the contribution assigned to primary-school enrollment increased from 37.33% in 2019 and 38.91% in 2020 to 57.99%, 60.35%, and 64.38% in 2021–2023. Contributions within the graduate and school-count models were less stable. Graduate contributions were not estimable in 2019–2020 because all corresponding coefficients were shrunk to zero, while the school-count models exhibited negative cross-validated R^2^ values throughout the study period. These decomposed results were therefore treated as exploratory.

**TABLE 2:** Relative SHAP contribution rates in exploratory decomposed Lasso models, 2019–2023.

Predictor domain	Subcomponent	2019	2020	2021	2022	2023
Industrial structure	Tertiary industry	67.48%	100.00%	96.41%	100.00%	100.00%
Industrial structure	Secondary industry	30.85%	0.00%	0.00%	0.00%	0.00%
Industrial structure	Primary industry	1.66%	0.00%	3.59%	0.00%	0.00%
Enrollment by school level	Regular secondary schools	46.18%	46.24%	42.01%	39.65%	10.21%
Enrollment by school level	Primary schools	37.33%	38.91%	57.99%	60.35%	64.38%
Enrollment by school level	Senior secondary schools	16.49%	14.85%	0.00%	0.00%	25.41%
Graduates by school level	Regular secondary schools	NE	NE	46.55%	46.55%	46.44%
Graduates by school level	Primary schools	NE	NE	33.43%	35.66%	38.07%
Graduates by school level	Senior secondary schools	NE	NE	20.02%	17.79%	15.49%
Number of schools by level	Senior secondary schools	100.00%	57.59%	100.00%	100.00%	100.00%
Number of schools by level	Regular secondary schools	0.00%	24.43%	0.00%	0.00%	0.00%
Number of schools by level	Primary schools	0.00%	17.98%	0.00%	0.00%	0.00%

NE indicates that all coefficients were shrunk to zero. These decomposed analyses are exploratory because several models had severe multicollinearity and weak or negative cross-validated R^2^.

Overall, Lasso provided the strongest predictive performance for the available prefecture-level datasets. Within the annual Lasso models, predictive importance shifted from economic-demographic indicators in 2019–2020 toward education-related indicators in 2021–2023. This pattern represents a redistribution of shared predictive information within the Lasso framework and should not be interpreted as a change in independent effects.

## 4 Discussion

### 4.1 Spatiotemporal patterns and geographic inequalities

This study identified persistent geographic inequalities in the dispensed volume of methylphenidate hydrochloride extended-release tablets across Jiangsu Province from 2019 to 2023. Dispensing was consistently higher in Southern Jiangsu, particularly in areas within Nanjing, Suzhou, and Wuxi, whereas Northern Jiangsu contained more county-level units with zero or low recorded volumes. Formal regional comparisons confirmed that this north–south contrast was not merely a visual pattern: Southern Jiangsu generally had the highest values, Northern Jiangsu the lowest, and Central Jiangsu occupied an intermediate position. These differences should not be attributed solely to geographic variation in ADHD prevalence. Medication dispensing reflects several sequential processes, including symptom recognition, referral, clinical diagnosis, physician availability, prescribing capacity, medication supply, and treatment continuity. Economically developed areas may have denser specialist services, stronger referral networks, greater public awareness, and fewer barriers to care. Accordingly, higher dispensing in Southern Jiangsu may partly reflect greater diagnostic and treatment accessibility, whereas low dispensing in Northern Jiangsu may represent lower clinical need, restricted access, or unmet treatment demand. This interpretation is consistent with studies linking psychotropic medication use to socioeconomic conditions, healthcare accessibility, and service organization (Brauer et al., 2021; Zhang et al., 2022; Dijkstra et al., 2013).

The decline in zero-volume areas and expansion of higher-volume categories suggest that medication dispensing became more geographically widespread. However, increasing tablet volume cannot distinguish improved access from changes in diagnosis, prescribing practice, treatment duration, or pharmaceutical supply. The nonsignificant Global Moran’s I results indicate that absolute tablet volumes did not form a uniform province-wide clustered pattern. Nevertheless, Gi* identified localized high-value concentrations, while no statistically significant low-value clusters were detected. This suggests that geographic inequalities were manifested through localized concentrations of high dispensing rather than through simultaneous hot- and cold-spot structures.

The study period also overlapped with the COVID-19 pandemic. School closures, disrupted outpatient services, altered referral pathways, and changes in prescription renewal may have affected ADHD diagnosis and medication access. The observed temporal changes should therefore not be interpreted as evidence of a discrete pandemic-related causal transition.

### 4.2 Socioeconomic and educational correlates

The predictive models indicated that methylphenidate dispensing was associated with an interrelated socioeconomic, demographic, and educational context. Urban population and GDP received relatively high predictive importance in the earlier years, consistent with evidence that stimulant use varies with population concentration, economic resources, and healthcare access (Grimmsmann and Himmel, 2021). Education-related indicators, particularly total enrollment and total school graduates, received greater predictive importance in some later annual Lasso models. Schools may contribute to ADHD recognition and referral because attention and behavioral difficulties frequently become visible in classroom settings. Previous studies have linked educational environments, parental involvement, socioeconomic inequality, and ADHD diagnosis or medication use (Owens, 2021; Khan and Hasan, 2025; Pearce et al., 2024).

Nevertheless, the education variables used here were aggregate indicators of school-system scale. They did not directly measure academic pressure, teacher quality, referral behavior, or individual treatment need. Total graduates represented the combined number of graduates from primary, regular secondary, and senior secondary schools and should not be interpreted as a direct cause of medication use.

The apparent changes in annual feature rankings also require caution because the predictors were strongly correlated. SHAP values allocate predictive contributions within the fitted model, and their distribution may shift among correlated variables or across algorithms. The results therefore do not demonstrate that educational factors replaced economic factors as the principal drivers of medication dispensing. A more defensible conclusion is that dispensing was associated with a combination of population scale, economic capacity, educational-system characteristics, and unmeasured healthcare conditions.

Exploratory decomposition suggested that tertiary-industry and some primary-school indicators contained relatively greater predictive information in certain years. These patterns are compatible with service-sector concentration and the childhood epidemiology of ADHD (Brauer et al., 2021; Raman et al., 2018), but they do not establish independent sectoral or school-level effects.

### 4.3 Methodological implications and limitations

Combining spatial analysis with regularized and explainable machine learning provided complementary perspectives on geographic inequalities and contextual associations. Lasso constrained model complexity in a small dataset with correlated predictors, while Random Forest, Gradient Boosting, and XGBoost were used to assess comparative predictive performance. SHAP further summarized the contribution of individual features to fitted predictions (Atias et al., 2025; Madakkatel et al., 2021; Wu et al., 2022; Yang et al., 2026). However, SHAP explains model behavior rather than causal mechanisms. Its values should be interpreted as model-specific predictive contributions, particularly because the annual models included only 13 prefecture-level cities and the predictors were highly correlated.

Several additional limitations should be noted. First, the ecological design prevents inference at the patient, family, school, or prescriber level. Second, tablet volume does not measure diagnosed prevalence, patient numbers, dosage, adherence, treatment duration, or prescribing appropriateness. Third, direct measures of physician supply, specialist services, diagnostic capacity, medication availability, teacher quality, and school referral practices were unavailable. Fourth, the small annual sample limited the stability of feature rankings and prevented reliable region-specific machine-learning models. Finally, local cluster classifications were sensitive to the spatial-weight specification. Future studies should integrate prescription- or patient-level records with age-specific population denominators and explicit indicators of healthcare capacity.

### 4.4 Policy implications

Policy responses should be differentiated by regional context. In Northern Jiangsu, persistently low dispensing and the high proportion of zero-volume areas warrant evaluation of possible access barriers and unmet diagnostic or treatment needs. Relevant measures include strengthening pediatric and child mental-health capacity in county hospitals, improving referral networks, supporting telemedicine consultation, and assessing medication-supply continuity. In Southern Jiangsu, persistently high volumes justify continued monitoring of diagnostic procedures, prescribing quality, treatment duration, and follow-up. High aggregate volume should not, however, be interpreted as evidence of overuse without patient- or prescription-level information. Central Jiangsu generally showed intermediate but changing patterns and may require surveillance for emerging service demand. Provincial monitoring should combine absolute tablet volumes, annual changes, zero-volume proportions, and localized spatial patterns, rather than applying a single province-wide threshold.

Health–education collaboration should focus on appropriate recognition and referral rather than treating school-system indicators as direct proxies for medication need. Schools can support early identification and family communication, but diagnosis and prescribing decisions should remain under qualified clinical supervision. Given concerns regarding stimulant prescribing and misuse, monitoring should ultimately link dispensing data with diagnosis, prescriptions, follow-up, and healthcare-resource information (Han et al., 2025; McCabe et al., 2023).

## 5 Conclusion

This study examined the spatiotemporal distribution and contextual correlates of methylphenidate hydrochloride extended-release tablet dispensing in Jiangsu Province from 2019 to 2023 by combining spatial analysis with interpretable machine-learning methods. The main conclusions are as follows:

1. The proportion of county-level units with zero recorded tablet volume decreased from approximately 52% in 2019 to 36% in 2023, while the proportion with annual volumes exceeding 20,000 tablets increased from 7% to 20%. These changes indicate broader dispensing coverage and an increasing number of areas entering higher-volume categories.
2. Southern Jiangsu consistently exhibited the highest dispensed volumes, Northern Jiangsu the lowest, and Central Jiangsu generally occupied an intermediate position. Formal regional comparisons confirmed significant differences among the three regions throughout the study period. Getis–Ord Gi* analysis identified localized high-value concentrations mainly in Southern and parts of Central Jiangsu; however, these local patterns should not be interpreted as evidence of uniform province-wide spatial clustering.
3. The spatial distribution expanded while its geographic center remained comparatively stable. The east–west standard deviation increased from 197.80 km to 213.12 km, the north–south standard deviation increased from 62.16 km to 71.04 km, and the standard deviational ellipse area increased from 38,626 km^2^ to 47,563 km^2^. Despite this expansion, the weighted centroid remained in south-central Jiangsu and showed only limited annual movement.
4. Methylphenidate dispensing was associated with an interrelated socioeconomic, demographic, and educational context. Urban population and GDP received relatively high predictive importance in the earlier annual Lasso models, whereas enrollment and total school graduates received greater importance in some later models. They instead indicate that regional dispensing patterns were jointly associated with population scale, economic capacity, school-system characteristics, and other unmeasured contextual conditions.

These findings support geographically differentiated surveillance: low-volume areas require assessment of possible access barriers and unmet need, whereas high-volume areas require review of prescribing quality and treatment follow-up. Future monitoring should integrate dispensing, diagnostic, prescription, healthcare-resource, and patient follow-up data.

## Data Availability

The raw data supporting the conclusions of this article will be made available by the authors, without undue reservation.
