# Supplementary material for: Spatial inequalities and contextual correlates of methylphenidate dispensing in Jiangsu Province, China
Source: Front Pharmacol. 2026 Aug 26;17:1926315. doi: 10.3389/fphar.2026.1926315 (PMC13553215; doi:10.3389/fphar.2026.1926315)
Supplement: Supplementary file 1 [file Supplementaryfile1.docx]

## Supplementary Table S1 **Regional heterogeneity in county-level methylphenidate dispensed tablet volume across Southern, Central, and Northern Jiangsu, 2019–2023**

### Panel A. Regional descriptive statistics and overall Kruskal–Wallis tests

| Year | Region | N | Mean (SD), tablets | Median (IQR), tablets | Zero-volume units, n (%) | Total tablets | Kruskal–Wallis H | p-value |
| --- | --- | --- | --- | --- | --- | --- | --- | --- |
| 2019 | Southern Jiangsu | 39 | 19,331.5 (59,597.3) | 300.0 (9,525.0) | 15 (38.5%) | 753,930 | 10.725 | 0.0047 |
|  | Central Jiangsu | 19 | 4,106.1 (11,973.4) | 30.0 (1,987.5) | 8 (42.1%) | 78,015 | — | — |
|  | Northern Jiangsu | 37 | 2,168.1 (10,623.7) | 0.0 (30.0) | 26 (70.3%) | 80,220 | — | — |
| 2020 | Southern Jiangsu | 39 | 22,730.0 (66,887.2) | 1,050.0 (12,787.5) | 12 (30.8%) | 886,470 | 13.991 | 0.0009 |
|  | Central Jiangsu | 19 | 10,228.4 (37,540.8) | 60.0 (1,387.5) | 8 (42.1%) | 194,340 | — | — |
|  | Northern Jiangsu | 37 | 2,733.6 (10,785.6) | 0.0 (90.0) | 26 (70.3%) | 101,145 | — | — |
| 2021 | Southern Jiangsu | 39 | 39,906.2 (113,923.4) | 3,060.0 (25,342.5) | 6 (15.4%) | 1,556,340 | 21.264 | <0.0001 |
|  | Central Jiangsu | 19 | 12,790.3 (46,884.0) | 300.0 (2,002.5) | 6 (31.6%) | 243,015 | — | — |
|  | Northern Jiangsu | 37 | 6,083.9 (26,994.4) | 0.0 (180.0) | 27 (73.0%) | 225,105 | — | — |
| 2022 | Southern Jiangsu | 39 | 45,344.2 (118,488.4) | 2,970.0 (35,287.5) | 6 (15.4%) | 1,768,425 | 19.248 | <0.0001 |
|  | Central Jiangsu | 19 | 17,343.2 (61,027.8) | 255.0 (3,412.5) | 7 (36.8%) | 329,520 | — | — |
|  | Northern Jiangsu | 37 | 8,300.3 (35,893.9) | 0.0 (420.0) | 24 (64.9%) | 307,110 | — | — |
| 2023 | Southern Jiangsu | 39 | 44,450.8 (116,156.3) | 4,650.0 (34,267.5) | 6 (15.4%) | 1,733,580 | 18.457 | <0.0001 |
|  | Central Jiangsu | 19 | 17,606.8 (61,403.7) | 465.0 (3,382.5) | 5 (26.3%) | 334,530 | — | — |
|  | Northern Jiangsu | 37 | 8,224.5 (34,656.6) | 0.0 (750.0) | 23 (62.2%) | 304,305 | — | — |

Panel B. Pairwise Mann–Whitney U tests with Holm adjustment

| Year | Regional comparison | Mann–Whitney U | Raw p-value | Holm-adjusted p-value |
| --- | --- | --- | --- | --- |
| 2019 | Southern vs. Central Jiangsu | 417.5 | 0.4263 | 0.4263 |
|  | Southern vs. Northern Jiangsu | 1,004.0 | 0.0014 | **0.0042** |
|  | Central vs. Northern Jiangsu | 457.0 | 0.0392 | 0.0783 |
| 2020 | Southern vs. Central Jiangsu | 458.0 | 0.1411 | 0.1411 |
|  | Southern vs. Northern Jiangsu | 1,051.5 | 0.0003 | **0.0008** |
|  | Central vs. Northern Jiangsu | 449.5 | 0.0555 | 0.1110 |
| 2021 | Southern vs. Central Jiangsu | 476.0 | 0.0806 | 0.0806 |
|  | Southern vs. Northern Jiangsu | 1,138.0 | <0.0001 | **<0.0001** |
|  | Central vs. Northern Jiangsu | 480.0 | 0.0130 | **0.0260** |
| 2022 | Southern vs. Central Jiangsu | 493.0 | 0.0421 | 0.0841 |
|  | Southern vs. Northern Jiangsu | 1,122.5 | <0.0001 | **<0.0001** |
|  | Central vs. Northern Jiangsu | 448.5 | 0.0669 | 0.0841 |
| 2023 | Southern vs. Central Jiangsu | 479.0 | 0.0726 | 0.0726 |
|  | Southern vs. Northern Jiangsu | 1,110.5 | <0.0001 | **<0.0001** |
|  | Central vs. Northern Jiangsu | 472.5 | 0.0258 | 0.0516 |

Note. Southern Jiangsu comprised Nanjing, Wuxi, Changzhou, Suzhou, and Zhenjiang; Central Jiangsu comprised Nantong, Yangzhou, and Taizhou; and Northern Jiangsu comprised Xuzhou, Lianyungang, Huai’an, Yancheng, and Suqian. These regions contained 39, 19, and 37 county-level analytical units, respectively.Values represent annual county-level dispensed tablet volumes. SD, standard deviation; IQR, interquartile range. Overall regional differences were evaluated using Kruskal–Wallis tests. When the overall test was significant, pairwise regional comparisons were conducted using Mann–Whitney U tests with Holm adjustment. Southern, Central, and Northern Jiangsu contained 39, 19, and 37 county-level analytical units, respectively.

**Supplementary Figure S1. Global Moran’s I for original county-level dispensed tablet volumes under alternative spatial-weight specifications, 2019–2023.**

Global Moran's I for original tablet counts. Queen contiguity was the primary specification; Rook contiguity and KNN6 were sensitivity specifications.

**Supplementary Table S2. Global Moran’s I for original county-level dispensed tablet volumes under alternative spatial-weight specifications, 2019–2023.**

| **Year** | **Weight** | **Moran's I** | **Expected I** | **Permutation z** | **Permutation p** | **Interpretation** |
| --- | --- | --- | --- | --- | --- | --- |
| 2019 | Queen | -0.019 | -0.011 | -0.154 | 0.845 | Not significant |
| 2019 | Rook | -0.020 | -0.011 | -0.161 | 0.851 | Not significant |
| 2019 | KNN6 | -0.034 | -0.011 | -0.567 | 0.460 | Not significant |
| 2020 | Queen | -0.013 | -0.011 | -0.069 | 0.966 | Not significant |
| 2020 | Rook | -0.014 | -0.011 | -0.096 | 0.953 | Not significant |
| 2020 | KNN6 | -0.027 | -0.011 | -0.376 | 0.697 | Not significant |
| 2021 | Queen | -0.011 | -0.011 | -0.015 | 0.991 | Not significant |
| 2021 | Rook | -0.012 | -0.011 | -0.033 | 0.981 | Not significant |
| 2021 | KNN6 | -0.023 | -0.011 | -0.274 | 0.782 | Not significant |
| 2022 | Queen | -0.008 | -0.011 | 0.057 | 0.973 | Not significant |
| 2022 | Rook | -0.009 | -0.011 | 0.039 | 0.990 | Not significant |
| 2022 | KNN6 | -0.027 | -0.011 | -0.370 | 0.728 | Not significant |
| 2023 | Queen | -0.009 | -0.011 | 0.034 | 0.983 | Not significant |
| 2023 | Rook | -0.010 | -0.011 | 0.017 | 0.995 | Not significant |
| 2023 | KNN6 | -0.027 | -0.011 | -0.386 | 0.736 | Not significant |

**Supplementary Table S3. Getis-Ord Gi* classification stability**

| **Year** | **Comparison** | **Exact agreement** | **Hot-spot Jaccard** | **Queen hot spots** | **Alternative hot spots** | **Queen cold spots** | **Alternative cold spots** |
| --- | --- | --- | --- | --- | --- | --- | --- |
| 2019 | Queen vs Rook | 1.000 | 1.000 | 8 | 8 | 0 | 0 |
| 2019 | Queen vs KNN6 | 0.853 | 0.438 | 8 | 15 | 0 | 0 |
| 2020 | Queen vs Rook | 1.000 | 1.000 | 9 | 9 | 0 | 0 |
| 2020 | Queen vs KNN6 | 0.863 | 0.333 | 9 | 11 | 0 | 0 |
| 2021 | Queen vs Rook | 1.000 | 1.000 | 9 | 9 | 0 | 0 |
| 2021 | Queen vs KNN6 | 0.863 | 0.333 | 9 | 11 | 0 | 0 |
| 2022 | Queen vs Rook | 1.000 | 1.000 | 9 | 9 | 0 | 0 |
| 2022 | Queen vs KNN6 | 0.874 | 0.333 | 9 | 11 | 0 | 0 |
| 2023 | Queen vs Rook | 1.000 | 1.000 | 8 | 8 | 0 | 0 |
| 2023 | Queen vs KNN6 | 0.842 | 0.467 | 8 | 14 | 0 | 0 |

**Note.** Cold-spot counts were zero under all raw-count specifications, so the cold-spot comparison was not informative. The stability interpretation therefore focuses on overall classification agreement and hot-spot Jaccard similarity.

Supplementary Figure S2. Standardized prefecture-level values of annual dispensed tablet volume and the five primary predictors, 2019–2023.

Supplementary Figure S3. Annual Pearson and Spearman correlation matrices for the five primary prefecture-level predictors, 2019–2023. The upper row presents Pearson correlation coefficients, and the lower row presents Spearman rank-correlation coefficients.
